# The involvement of the phenylpropanoid and jasmonate pathways in methyl jasmonate-induced soft rot resistance in kiwifruit (*Actinidia chinensis*)

**DOI:** 10.3389/fpls.2022.1097733

**Published:** 2022-12-16

**Authors:** Shucheng Li, Liuhua Xiao, Ming Chen, Qing Cao, Zhenyu Luo, Naihui Kang, Mingshu Jia, Jinyin Chen, Miaolian Xiang

**Affiliations:** ^1^ College of Agronomy, Jiangxi Agricultural University, Nanchang, China; ^2^ Jiangxi Key Laboratory for Postharvest Technology and Nondestructive Testing of Fruits & Vegetables, Jiangxi Agricultural University, Nanchang, China; ^3^ Scientific Research Division, Nanchang Institute of Technology, Nanchang, China

**Keywords:** kiwifruit, methyl jasmonate, Botryosphaeria dothidea, phenylpropanoid pathway, jasmonate pathway

## Abstract

*Botryosphaeria dothidea* is a major postharvest causal agent of soft rot in kiwifruit. Methyl jasmonate (MeJA) is an important plant hormone that participates as a plant defense against pathogens from a signal molecule. However, the impact and regulatory mechanism of MeJA on the attenuation of kiwifruit fungal decay remains unknown. This work investigated the effects of exogenous MeJA on the enzyme activity, metabolite content and gene expression of the phenylpropanoid and jasmonate pathways in kiwifruit. The results revealed that MeJA inhibited the expansion of *B. dothidea* lesion diameter in kiwifruit (*Actinidia chinensis* cv. ‘Hongyang’), enhanced the activity of enzymes (phenylalanine ammonia lyase, cinnamate 4-hydroxylase, 4-coumarate: coenzyme A ligase, cinnamyl alcohol dehydrogenase, peroxidase and polyphenol oxidase), and upregulated the expression of related genes (*AcPAL*, *AcC4H*, *Ac4CL*, and *AcCAD*). The accumulation of metabolites (total phenolics, flavonoids, chlorogenic acid, caffeic acid and lignin) with inhibitory effects on pathogens was promoted. Moreover, MeJA enhanced the expression of *AcLOX*, *AcAOS*, *AcAOC*, *AcOPR3*, *AcJAR1*, *AcCOI1* and *AcMYC2* and reduced the expression of *AcJAZ*. These results suggest that MeJA could display a better performance in enhancing the resistance of disease in kiwifruit by regulating the phenylpropanoid pathway and jasmonate pathway.

## 1 Introduction

Kiwifruit (*Actinidia chinensis*) is a fruit with high nutritional and economic value from its pleasurable taste and richness in bioactive compounds, including vitamin C, carotenoids and tocopherols (Alim et al., 2019). However, kiwifruit often suffers mechanical damage from the harvesting and transportation of the fruit that causes a range of physiological and pathological reactions during storage. The common pathogens that cause kiwifruit diseases postharvest include *Botrytis cinerea* (Michailides and Elmer, 2000), *Penicillium expansum* (Wang et al., 2015a), and *Botryosphaeria dothidea* (Zhou et al., 2015). Postharvest soft rot caused by *B. dothidea* is the most important disease of kiwifruit in Jiangxi Province, China. Soft rot mainly affects the quality of the fruit and generates severe economic losses for kiwifruit production. In recent years, the increasing interest in issues that stem from pesticide residue has advanced the rapid development of disease control measures for alternative fungicides (Carlos et al., 2019). Induction of defense resistance by abiotic and biotic factors has been a primary research topic to control postharvest diseases of fruit and vegetables, and many inducers of natural disease resistance have been increasingly applied in production (Chen et al., 2021).

Methyl jasmonate (MeJA) is a regulator that is present in plants and acts as a signaling molecule by participating in a variety of physiological processes, as well as playing a significant role in plant disease resistance and defense responses (Yu et al., 2019). MeJA treatment can ultimately enhance the ability of plants to defend against adverse stress by inducing the signaling pathways of the plant physiological metabolism and regulating the expression of disease resistance genes. For example, exogenous MeJA treatment improved the disease resistance of Chinese bayberries (*Myrica rubra* Seib & Zucc.) (Wang et al., 2009), avocado (*Persea americana* Mill.) (Glowacz et al., 2017) and blueberry (*Vaccinium ashei* Reade) (Wang et al., 2020a) by enhancing the activity of antioxidant systems and pathogenesis-related proteins. It has also been found that the transcription factor *MYC2* is a critical regulator in the jasmonic acid-based hormone regulatory network, which improves cold tolerance and resistance to gray mold in tomato by regulating the defense of enzyme activity and increasing the accumulation of proline and phytoalexin (Min et al., 2018; Min et al., 2021). In addition, MeJA has been able to induce crucial genes (lipoxygenase (*LOX*), allene oxide synthase (*AOS*) and 12-oxophytodienoate reductase 3 (*OPR3*)) of jasmonate pathway that increased their expression levels, thus enhancing disease resistance in peach (Ji et al., 2021) and sweet cherry fruit (Pan et al., 2022). Furthermore, MeJA could effectively improve the antioxidant system of fruit and vegetables and therefore delay the decline in the quality of fruit and vegetables and extended the storage time, including peach (*Prunus persica* L.) (Meng et al., 2009), blueberry (Wang et al., 2019) and lemon (*Citrus limon* (L.) Burm. F.) (Serna-Escolano et al., 2021).

Previous studies conducted by our research team have confirmed that 0.1 mmol L^-1^ MeJA activates the reactive oxygen metabolism in kiwifruit (*Actinidia deliciosa* cv. Jinkui) by inducing the expression of defense genes, thereby enhancing the antioxidant capacity of fruit, which improves the resistance to soft rot and effectively maintains fruit quality (Pan et al., 2020). However, no studies are available on the effects of MeJA on the phenylpropanoid pathway and jasmonate pathway during the resistance to disease in kiwifruit. The purpose of this research was to investigate the effects of MeJA on the expression of key enzyme activities and metabolites of the phenylpropanoid pathway, as well as the expression levels of jasmonate pathway genes in kiwifruit. This work will contribute to the further understanding of the mechanisms behind the effect of MeJA in the process of kiwifruit resistance to the pathogen *B. dothidea*.

## 2 Materials and methods

### 2.1 Fruit

*Actinidia chinensis* cv. ‘Hongyang’ is one of the main kiwifruit varieties cultivated in Jiangxi province, and the whole genome sequencing has been completed (Yue et al., 2020). ‘Hongyang’ kiwifruit was selected as the tested fruit based on its high sensitivity to *B. dothidea* (Wang et al., 2020b). At maturity, kiwifruit were harvested from an orchard (28.71°N, 115.38°E, Fengxin County, Jiangxi Province, China) when the content of the soluble solids reached 7.0-7.5% (n = 18) on Aug. 25, 2021. The collected fruit were immediately transported to the laboratory; then, fruit of uniform size, without symptoms of mechanical injury and plant disease, were selected for the experiment.

### 2.2 Pathogen

*B. dothidea* was previously isolated from decayed kiwifruit and then stored at -80°C. It was cultured on a potato dextrose agar medium (28 ± 1°C, 90 ± 5% humidity) one week prior to the experiment.

### 2.3 Fruit treatment

The kiwifruits were randomly divided into three groups as follows: (i) the inoculation group, where the fruit did not undergo MeJA treatment but did receive an inoculation of *B. dothidea*; (ii) the MeJA+inoculation group, in which the fruit underwent MeJA treatment and received an inoculation of *B. dothidea*; and (iii) the control group, the fruit was exposed to the air, and there was no MeJA treatment and inoculation. When performing the group ii treatment, liquid MeJA (Sigma–Aldrich, USA) was dropped onto sterile filter paper and quickly placed into sealed boxes (80 L) that contained the kiwifruit, with the final concentration was 0.1 mmol L^-1^ based on the volatility of MeJA. The fruit of the group i were treated with equal amounts of distilled water using the same method. Each group was repeated three times with 120 fruit per repeated experimental procedure. All fruit were treated at 20 ± 1°C for 24 h and then exposed to a clean bench for 30 min at 20 ± 1°C.

The surface of the fruit was disinfected using a wipe with 75% ethanol, the epidermis was punctured with a sterile inoculation needle at the equator of the fruit, and 30 µL of spore suspension (1.0 × 10^6^ spores mL^-1^) was inoculated into the wound. The kiwifruits were arranged in fresh-keeping boxes (20 ± 1°C, 90%-95% humidity) for 8 days. The tissue annulus of 20 ± 5 mm that was around the lesion was collected daily, and these tissues were frozen with liquid nitrogen and then ground into a powder, and stored at -80°C. Twelve fruit per treatment group were randomly selected each time and the procedure was replicated three times.

### 2.4 Determination of disease lesion size

The diameter of the lesions on the fruit was measured with a Vernier scale (Shanghai Measuring & Cutting Tool Works Co., Ltd, China) using the cross method. Twelve fruit per treatment group were randomly selected for measurement each time and replicated three times. Induction effect = [(lesion diameter of group i – lesion diameter of group ii)/lesion diameter of group i] × 100%.

### 2.5 Determination of phenylpropanoid metabolism enzyme activity

Phenylalanine ammonia lyase (PAL) (EC 4.3.1.24) was determined using an assay kit (NJBI, Co., Ltd, China) to measured its absorbance at 290 nm, and the operating manual was strictly followed.

The enzyme activity assays for cinnamate 4-hydroxylase (C4H) (EC 1.14.14.91), 4-coumarate: coenzyme A ligase (4CL) (EC 6.2.1.12) and cinnamyl alcohol dehydrogenase (CAD) (EC 1.1.1.195) were performed as previously described (Liu et al., 2014; Takshak and Agrawal, 2014). Approximately 1.0 g of sample powder was mixed with 5 mL of 200 mmol L^-1^ Tris-HCl buffer (pH 7.5) including 100 mmol L^-1^ dithiothreitol and 25% (v/v) glycerol, then the supernatant was obtained after centrifugation at 9000 × g at 4°C. The C4H reaction system contained 2 mL of 50 mmol L^-1^ Tris-HCl buffer (containing 0.08 mmol L^-1^ NADPNa_2_, 0.6 mmol L^-1^ G-6-PNa_2_ and 8 mmol L^-1^ trans-cinnamic acid) and 0.8 mL of supernatant, and the absorbance value was measured at 340 nm after mixing. The absorbance of 4CL was measured at 333 nm by mixing 2 mL of 50 mmol L^-1^ Tris-HCl buffer (containing 0.2 mmol L^-1^ *p*-coumarate, 2.5 mmol L^-1^ MgCl_2_, 2.5 mmol L^-1^ ATP and 0.4 mmol L^-1^ CoA) with 0.5 mL of supernatant. A mixture of 2.5 mL of NADP (2.0 mmol L^-1^) and trans-cinnamic acid (1 mmol L^-1^) was added to 0.5 mL of supernatant, and the absorbance of CAD was measured at 340 nm after 30 min reaction at 37°C.

The enzyme activities of peroxidase (POD) (EC 1.11.1.7) and polyphenol oxidase (PPO) (EC 1.10.3.1) were measured by a previously described methodology (Li et al., 2019a). The enzyme extract was obtained by mixing 1.0 g of sample with 5 mL of 50 mmol L^-1^ phosphate buffer (pH 7.5, containing 2% (w/v) polyvinylpyrrolidone and 0.1% (v/v) Triton X-100) and centrifuging at 9000 × g for 20 min at 4°C. The POD reaction system consisted of 3.0 mL of 25 mmol L^-1^ guaiacol, 0.2 mL of 500 mmol L^-1^ H_2_O_2_ and 0.5 mL of enzyme extract, and the change in absorbance was detected at 470 nm. The PPO activity was detected at 420 nm by mixing 0.15 mL of enzyme extract, 2.0 mL of phosphate buffer (50 mmol L^-1^) and 0.5 mL of catechol (50 mmol L^-1^).

All enzyme activity was expressed as U kg^-1^ on the fresh weight basis. One unit of enzyme activity was equal to 0.01 increase per minute in the corresponding absorbance. All assays were repeated three times.

### 2.6 Determination of total phenolic, flavonoid and lignin contents

Lignin was assayed according to the description of Xu et al. (2019) with minor modification. The sample powder (1.0 g) was mixed in 5.0 mL of 95% ethanol and centrifuged at 9000 × g for 25 min at 4°C. The sediment was rinsed with 95% ethanol and ethanol: hexane (1: 2, v/v) respectively, and dried (65°C, 2 h). Then the sediment was dissolved with 1.0 mL of bromoacetyl bromide-acetic acid (25%, w/v), incubated for 30 min at 70°C, and the reaction was terminated by adding 1.5 mL of NaOH (2.0 mol L^-1^). The results were expressed as OD_280_ kg^-1^ on the fresh weight basis.

The total phenolic and total flavonoid contents were assayed as previously described by Wei et al. (2017). The extracts were obtained by mixing 2.5 g of sample with 10 mL of methanol (70%, v/v), shaking for 1 h and then centrifuging at 9000 × g for 20 min at 4°C. The absorbance of total phenols and flavonoids was read using UV-VIS spectrophotometer (Purkinje General Instrument Co., Ltd, China) at 760 nm and 510 nm, respectively. The total phenolic content was calculated using a gallic acid standard curve, and the flavonoid content was calculated based on the rutin standard curve. The contents were all expressed as g kg^-1^ on the fresh weight basis. The assay was performed in three replicates.

### 2.7 Determination of phenolic acid content

The contents of chlorogenic acid, caffeic acid and *p*-coumaric acid in the kiwifruit pulp were determined. The methodologies for extraction and assay were as previously discussed in Zhang et al. (2010), with some slight modifications. Phenolic compounds were analyzed using a low-pressure gradient HPLC Shimadzu system that was equipped with a photodiode array detector (Model: LC-2030 Plus, Shimadzu Corporation, Kyoto, Japan). An ODS-100-V 5-μm 4.6-mm I.D.× 25-cm column (Tosoh Corporation, Tokyo, Japan) was used in the separation procedure. Mobile phase A contained 2% acetic acid in the water (v/v), and phase B was chromatographically pure methanol. The detection wavelength was set to 320 nm. The content were all expressed as mg kg^-1^ on the fresh weight basis. Triplicate determinations were performed.

### 2.8 RNA extraction and cDNA synthesis

The total RNA of the kiwifruit was obtained using an RNAprep Pure Plant Kit (Tiangen, Co., Ltd, China) according to the product instructions. The quality and concentration of RNA were detected using 1% agarose gel electrophoresis and a nucleic acid analyzer (Biochrom Ltd, Cambridge, UK). A kit (Yeasen, Co., Ltd, China) was used to reverse-transcribe the RNA into cDNA.

### 2.9 Gene expression analysis

The gene expression level was detected by RT–qPCR using TB Green^®^ Premix Ex Taq (Takara Bio Inc., Japan). The sample at 0 d was used as internal calibrator, and the *AcActin* was used as the house-keeping gene. The primer sequences are shown in **Table S1**. The gene expression was calculated according to the 2^−ΔΔCt^ method (Livak and Schmittgen, 2001). Each experiment being performed in triplicate.

### 2.10 Statistics

The data were processed and analyzed using SPSS 22.0 (SPSS Inc., USA). The significance of differences between groups was determined using Duncan’s multiple range tests at the 5% level. The figures were drawn using Excel 2016 (Microsoft, USA). Correlation analysis was performed by Origin 2022 (Origin Lab Co., USA) with Pearson’s correlations and two-sided tests.

## 3 Results

### 3.1 MeJA affects the lesion diameter in kiwifruit

**Figure 1:**
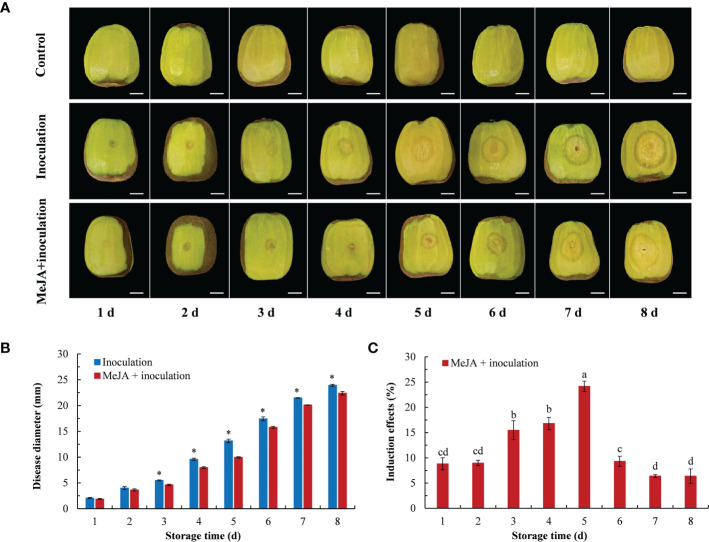
Effect of MeJA on diseases caused by *B. dothidea* in kiwifruit during storage time. **(A)** The symptoms of kiwifruits inoculated with *B. dothidea* in different treatment groups (Bar = 1 cm). **(B)** MeJA affects lesion diameter during storage time. **(C)** Induction effect of MeJA on kiwifruits. Bars shows standard deviation (SD, n=3). Letters and “*” indicate significant differences at *P* < 0.05 level among the treatments.

The effects from the different treatments inducing the resistance of kiwifruit to *B. dothidea* in the postharvest stage are shown in **Figure 1**. The lesion diameter gradually increased with increasing inoculation time (**Figure 1A**). After 3 d of inoculation, there were differences between the inoculation group and the MeJA+inoculation group, and the lesion diameter of the MeJA+inoculation group was smaller than that of the inoculation group (**Figure 1B**). The induction of 0.1 mmol L^-1^ MeJA treatment on kiwifruit resistance to soft rot showed a trend of first increasing and then decreasing (**Figure 1C**), with the best induction effect of 24.16% at 5 d after inoculation. This was followed by 3 d and 4 d after inoculation, for which the induction effects were 15.48% and 16.80%, respectively.

### 3.2 MeJA affects enzymatic activities of the phenylpropanoid pathway in kiwifruit

**Figure 2:**
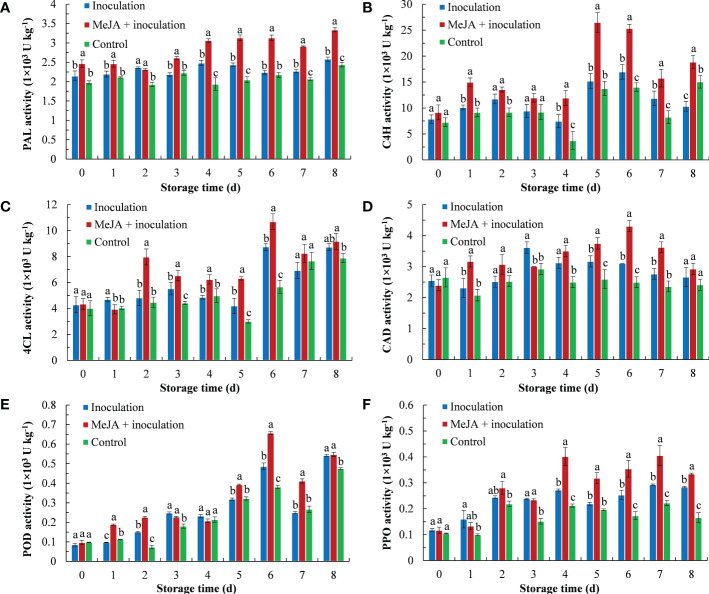
MeJA affected the activities of **(A)** PAL (phenylalanine ammonia lyase), **(B)** C4H (cinnamate 4-hydroxylase), **(C)** 4CL (4-coumarate: coenzyme A ligase), **(D)** CAD (cinnamyl alcohol dehydrogenase), **(E)** POD (peroxidase) and **(F)** PPO (polyphenol oxidase) in kiwifruit during storage time. Bars shows standard deviation (SD, n=3). Letters indicate significant differences at *P* < 0.05 level among the treatments.

MeJA improved the PAL enzyme activity of kiwifruit (**Figure 2A**), whereby an increasing trend of PAL activity was demonstrated with the extension of inoculation time. The PAL activity of the MeJA+inoculation group was higher than that of the inoculation group except for 2 d after inoculation.

As shown in **Figure 2B**, the C4H enzyme activity first increased and then decreased, and the MeJA-treated kiwifruit remained higher level during storage. The C4H enzyme activity peaked of 26.49 U kg^-1^ at 5 d after inoculation, which was 1.75 and 1.93 times higher than that of the inoculation and control groups, respectively.

The 4CL activity is shown in **Figure 2C**, and MeJA increased its activity from 2 d to 6 d. The 4CL activity of the MeJA-treated kiwifruit increased sharply at 2 d and was 1.66 times that of the inoculation group. The activity of the 4CL peaked at 6 d.

Over the course of 8 d after being treated with MeJA, the activity of CAD increased from 0 d to 6 d and then decreased in the following period (**Figure 2D**). The CAD activity of the MeJA+inoculation group showed the highest activity (4.31 U kg^-1^) at 6 d, which was 1.39 times that of the inoculation groups.

The process of POD activity is shown in **Figure 2E**. The POD activity of the MeJA+inoculation group increased sharply from 4 d to 6 d. From 6 d, the POD activity was 1.35 and 1.72 times that of the inoculation and control groups, respectively.

MeJA treatment increased the PPO activity of kiwifruit from 4 d to 8 d (**Figure 2F**). At 4 d of inoculation, the MeJA+inoculation group had the maximum fold difference in activity from the inoculation group, which was 1.66 times that of the inoculation group.

### 3.3 MeJA affects phenylpropanoid metabolites in kiwifruit

#### 3.3.1 MeJA affects the total phenolic, flavonoid and lignin contents in kiwifruit

**Figure 3:**
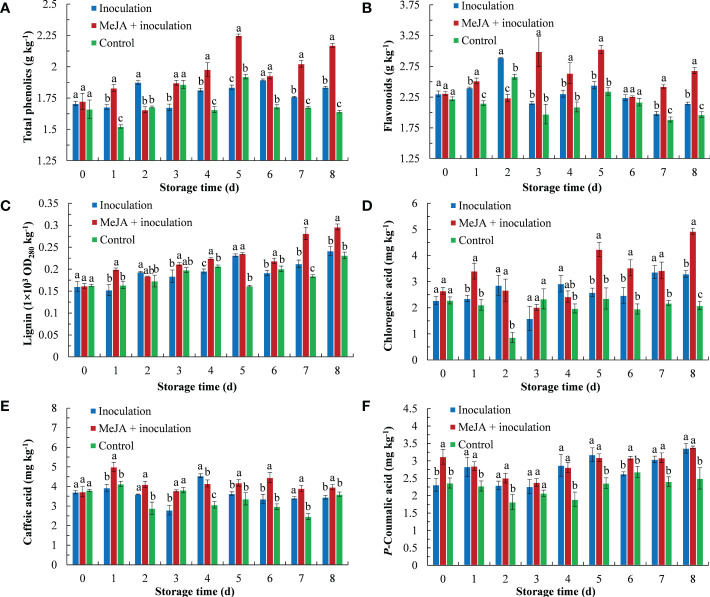
MeJA affected the content of **(A)** total phenolics, **(B)** flavonoids, **(C)** lignin, **(D)** chlorogenic acid, **(E)** caffeic acid and **(F)** *p*-coumaric acid in kiwifruit during storage time. Bars shows standard deviation (SD, n=3). Letters indicate significant differences at *P* < 0.05 level among the treatments.

The total phenolic content is shown in **Figure 3A**. MeJA increased the total phenolic content of kiwifruit, which was higher than the inoculation and control groups except from 2 d to 3 d. Its highest content peaked at 5 d, which was 1.22 times that of the inoculation group.

MeJA treatment maintained a high level of flavonoid content (**Figure 3B**). The maximum flavonoid content in MeJA-treated kiwifruit tissues was observed at 3 d and 5 d after treatment, which was 1.39 times and 1.24 times that of the inoculation group, respectively.

As storage time progressed, the lignification of fruit in the three groups gradually increased (**Figure 3C**). The lignin content of the three groups reached its maximum at 8 d, at which time the lignin content of the MeJA+inoculation group was 1.23 and 1.28 times that of the inoculation and control groups, respectively.

#### 3.3.2 MeJA affects the chlorogenic acid, caffeic acid and p-coumaric acid contents in kiwifruit

The chlorogenic acid content is shown in **Figure 3D**. Chlorogenic acid rapidly accumulated in the MeJA+inoculation group (3.41 mg kg^-1^) in comparison to the inoculation and control groups at 1 d. Chlorogenic acid of the MeJA+inoculation group exhibited its highest content at 8 d, which was 1.50 and 2.36 times that of the inoculation and control groups, respectively.

The content of caffeic acid in the MeJA+inoculation group increased sharply and peaked at 1 d, which was 1.27 times that of the inoculation group (**Figure 3E**). In addition, the MeJA+inoculation group was also higher than the inoculation and control groups from 5 d to 8 d.

The *p*-coumaric acid content in kiwifruit showed an obvious increase after inoculation with *B. dothidea* (**Figure 3F**). There were no differences between the MeJA+inoculation and inoculation groups except at 0 d and 6 d.

### 3.4 MeJA affects the expression of genes of phenylpropanoid and jasmonate pathway in kiwifruit

#### 3.4.1 Gene expression of the phenylpropanoid pathway

**Figure 4:**
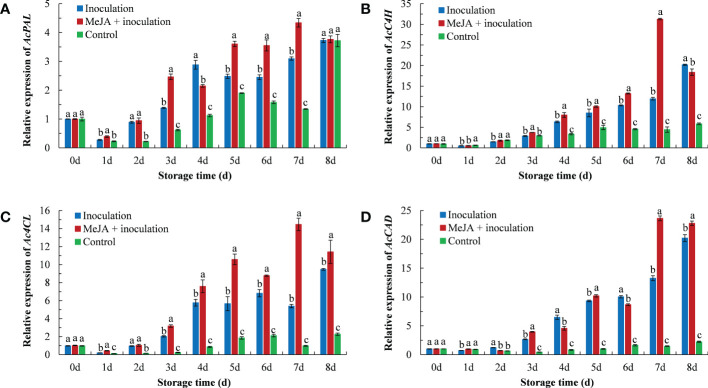
MeJA affected the gene expression of **(A)** *AcPAL*, **(B)** *AcC4H*, **(C)** *Ac4CL*, and **(D)** *AcCAD* in kiwifruit during storage time. Bars shows standard deviation (SD, n=3). Letters indicate significant differences at *P* < 0.05 level among the treatments.

The expression of phenylpropanoid-related genes in kiwifruit after the MeJA treatment is shown in **Figure 4**. The *AcPAL* expression in the MeJA+inoculation group began to rise on 1 d and peaked in the expression at 7 d (**Figure 4A**). The *AcPAL* expression was approximately 1.40 times that of the inoculation group and 3.22 times that of the control group in MeJA-treated kiwifruit tissues at 7 d.

The *AcC4H* expression in the MeJA+inoculation group reached a maximum at 7 d (**Figure 4B**), which was 2.62 and 7.06 times that of the inoculation and control groups, respectively. The control group maintained a lower expression level throughout the process.

In addition, the pattern of *Ac4CL* expression in kiwifruit was also similar to the result obtained for *AcC4H* (**Figure 4C**). The *Ac4CL* expression in the inoculation group was the highest at 8 d, and the *Ac4CL* expression in the MeJA+inoculation group peaked at 7 d.

As shown in **Figure 4D**, the *AcCAD* expression demonstrated an increasing trend with increasing inoculation time. The maximum expression of *AcCAD* in MeJA-treated kiwifruit was at 7 d, which was 1.78 and 16.00 times that of the inoculation and control groups, respectively.

#### 3.4.2 Gene expression of the jasmonate pathway

**Figure 5:**
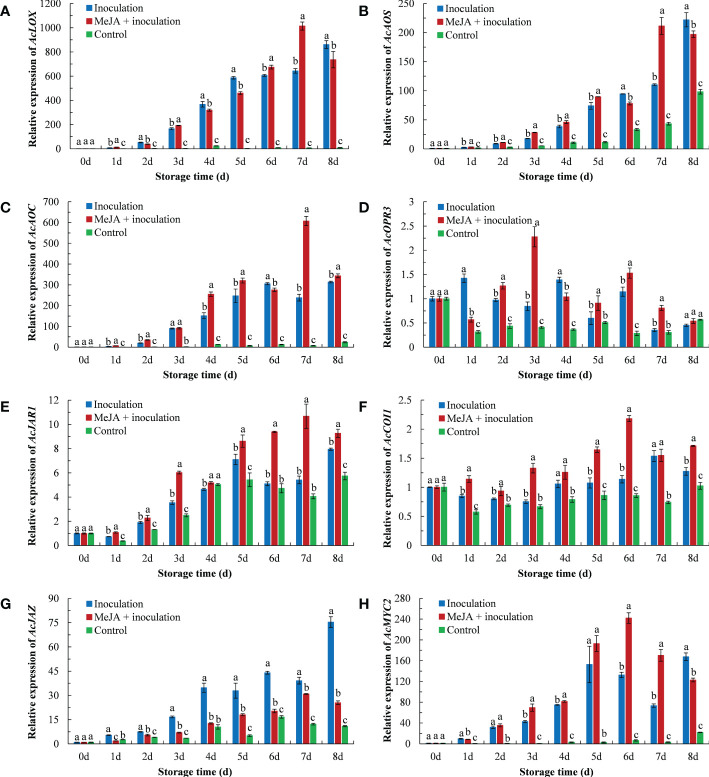
MeJA affected the gene expression of **(A)** *AcLOX*, **(B)** *AcAOS*, **(C)** *AcAOC*, **(D)** *AcOPR3*, **(E)** *AcJAR1*, **(F)** *AcCOI1*, **(G)** *AcJAZ*, and **(H)** *AcMYC2* in kiwifruit during storage time. Bars shows standard deviation (SD, n=3). Letters indicate significant differences at *P* < 0.05 level among the treatments.

As shown in **Figure 5**, the inoculation with *B. dothidea* in kiwifruit activated the expression of crucial genes of the jasmonate pathway, which was obviously higher than that in the control group. Compared to the inoculation group, the MeJA+inoculation group upregulated the expression of *AcLOX*, *AcAOS*, *AcAOC*, *AcOPR3*, *AcJAR1*, *AcCOI1* and *AcMYC2* during the majority of the storage period. The *AcLOX* expression in the MeJA+inoculation group peaked at 7 d (**Figure 5A**), which were 1.58 and 137.79 times that in the inoculation and control groups, respectively. The overall pattern of the *AcAOS* expression in MeJA-treated kiwifruit was similar to that of *AcLOX* (**Figure 5B**). The *AcAOS* expression reached its maximum at 7 d in the MeJA-treated kiwifruit. The *AcAOC* expression in MeJA-treated kiwifruit was the highest at 7 d (**Figure 5C**), which was 2.83 times that of the inoculation group. MeJA treatment enhanced *AcOPR3* expression in inoculated *B. dothidea* kiwifruit except at 1 d and 4 d (**Figure 5D**). Expression of *AcJAR1* was enhanced by MeJA treatment, and the maximum expression was at 7 d, which was 1.97 times that of the inoculated group (**Figure 5E**). The *AcCOI1* expression in the MeJA+inoculation group peaked at 6 d (**Figure 5F**), which was 1.91 and 2.53 times that in the inoculation and control groups, respectively. Compared with the inoculation group, MeJA treatment induced a downregulation of *AcJAZ* throughout the storage period. The *AcJAZ* expression in the inoculation group peaked at 8 d (**Figure 5G**), which was 2.96 and 6.87 times that of the MeJA+inoculation and control groups, respectively. The expression of *AcMYC2* in MeJA-treated kiwifruit was upregulated from 3 d to 7 d (**Figure 5H**). The highest level of the *AcMYC2* expression in the MeJA+inoculation group occurred at 6 d, which was 1.83 times higher during this time than that in the inoculation group.

### 3.5 Correlation analysis

**Figure 6:**
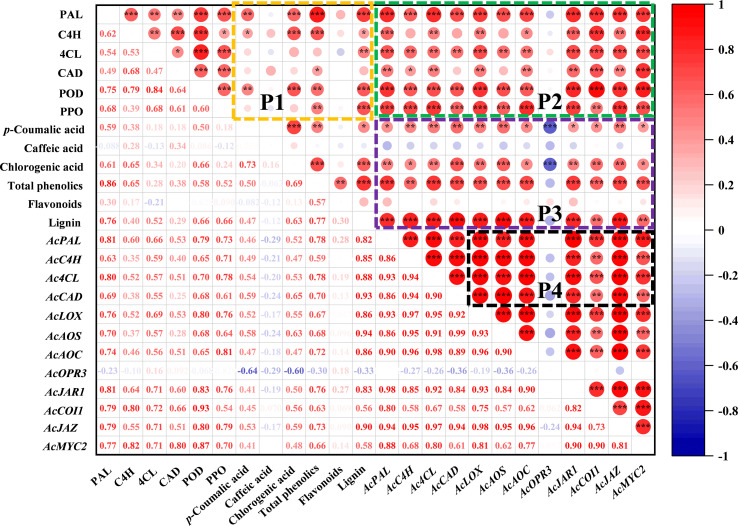
Correlation analysis between indexes of phenylpropanoid and jasmonate pathway in kiwifruit. The graph was plotted by the relative levels of all indicators in MeJA-treated kiwifruit during *B. dothidea* inoculation, based on the Pearson’s correlation coefficients obtained. The red and blue colors represent positive and negative correlations, respectively. *P < 0.05; **P < 0.01; ***P < 0.001.

The correlation analysis between indexes of phenylpropanoid and jasmonate pathway is shown in **Figure 6**. There are complex and varied correlations among the indicators in the P1 region. The accumulation of total phenolics was closely related to PAL (R^2^ = 0.86) and C4H (R^2^ = 0.65) enzymes. All six enzyme activities related to the phenylpropanoid pathway were positively correlated with lignin, especially PAL (R^2^ = 0.76), POD (R^2^ = 0.66) and PPO (R^2^ = 0.66). Moreover, *p*-coumaric acid was positively correlated with PAL (R^2^ = 0.59), and chlorogenic acid with PAL (R^2^ = 0.61), C4H (R^2^ = 0.65) and POD (R^2^ = 0.66). In P2 region, the enzyme activities of the phenylpropanoid pathway showed an overall positive correlation with gene expression. The correlation between metabolites and gene expression is reflected in P3, gene expression of phenylpropanoid and jasmonate pathway was positively correlated with most phenolic compounds, except for the *AcOPR3* with *p*-coumaric (R^2^ = -0.64) and chlorogenic (R^2^ = -0.60). There was a similar correlation between the expression trends of jasmonate-related genes and *AcPAL*, *AcC4H*, *Ac4CL*, *AcCAD* in P4 region.

## 4 Discussion

MeJA is an important regular of natural plant growth that acts as a signaling molecule in the plant metabolism network to stimulate the immune system of fruit and effectively improve the resistance of fruit against fungal infection (Wang et al., 2022). Previous studies have shown that exogenous MeJA treatment is beneficial in reducing pre-harvest potato (Yang et al., 2022) and post-harvest citrus (Guo et al., 2014) diseases. In addition, MeJA also has a direct inhibitory effect on pathogens. Wang et al. (2015b) found that 10 μmol L^-1^ MeJA could inhibit spore germination of *Penicillium expansum* through the use of *in vitro* experiments, and Tzortzakis et al. (2016) also confirmed that 44.8 μL L^-1^ MeJA could inhibit the growth of mycelium and spore production of gray mold. However, in contrast to the results of Yao and Tian (2005), mycelial growth and spore germination of *Monilinia fructicola* were not inhibited by 0.2 mmol L^-1^ MeJA. These results indicated that different pathogenic species have different sensitivities to MeJA. A previous study in our laboratory confirmed that the inhibitory effect of MeJA on the growth of *B. dothidea* mycelium was positively correlated with the concentration (Pan et al., 2019), and the results were the same as those reported by Li et al. (2019c). However, when it was applied to kiwifruit, 1 mmol L^-1^ MeJA treatment conversely reduced the effect of induction of fruit resistance (Pan et al., 2019). Therefore, MeJA acts as a signaling molecule to resist the infection of pathogens by activating the immune system of kiwifruit. In this study, 0.1 mmol L^-1^ MeJA treatment slowed the expansion of kiwifruit lesions, indicating that MeJA plays a critical role in enhancing the disease resistance of kiwifruit.

Phenylpropanoid pathway is an important secondary metabolic pathway in plants and has an essential role in plant disease resistance. Enzymes, including PAL, C4H, 4CL and CAD, in the phenylpropanoid pathway regulate the biosynthetic pathway of secondary metabolites and catalyze a range of reactions to generate substances with antimicrobial effects, such as phenolic acids, flavonoids, and lignin (Zhou et al., 2019). In the present study, MeJA enhanced the PAL, C4H, 4CL and CAD activities and their related gene expression in kiwifruit. There was a positive correlation between enzyme activity and gene expression. The results were similar to those of previous experiments conducted on blueberry (Wang et al., 2020a). POD and PPO are important catalytic enzymes for fruit that can participate in the synthesis of certain metabolites (e.g., hormones, phytoalexin and phenolics), and they enhance the system of defense, thereby inducing disease resistance in kiwifruit. POD can catalyze the polymerization of synthetic precursors of phenolic substances into lignin, which is deposited into the cell wall to defend against fungal infection. To further form toxic terpenoids, PPO can be used to catalyze phenolic substances, which have a direct inhibitory effect on pathogens (Ge et al., 2019). This study demonstrated that postharvest MeJA treatment increased the POD and PPO enzyme activities in kiwifruit, promoted the accumulation of lignin content, and enhanced the lignification of infected tissues. Similar results have been reported for muskmelon fruit that were treated with oxalic acid, whereby the accumulation of lignin strengthened the cell wall, and the histological structure as a preventive barrier was markedly strengthened (Deng et al., 2015). Rapid wound healing contributes to the maintenance of fruit quality and resistance to pathogens, and studies have shown that the phenylpropanoid pathway is an important pathway by which MeJA promotes wound healing in kiwifruit (Wei et al., 2021). The results of this experiment corroborated the important role of this pathway in the disease resistance process. Phenolics and flavonoids are not only antioxidant substances but also important defense substances in plants. They are able to damage the structure of the plants, and interfere with the physiological functions of pathogens, and induce plants to activate their own immune system, which is closely related to the resistance of plant disease (Zhang et al., 2020). Different elicitors had similar induction effects in various fruit and vegetables, such as pitaya treated with β-aminobutyric acid (Li et al., 2019a), kiwifruit treated with alginate oligosaccharide (Zhuo et al., 2022) and cherry tomato treated with melatonin (Li et al., 2019b), which improved the ability of fruit to withstand adversity stress by promoting the synthesis of phenolics. This study showed that exogenous MeJA treatment promoted the accumulation of total phenolics, flavonoids, lignin, chlorogenic acid and caffeic acid in kiwifruit and strengthened the defense capacity of the fruit. In addition, MeJA treatment also had no obvious effect on the *p*-coumaric acid content, and therefore it was speculated that the aromatic ring of *p*-coumaric acid had been hydroxylated and methylated to form its derivatives that played a critical role during the process (Heleno et al., 2015). Correlation analysis showed that the total phenolic, chlorogenic acid and *p*-coumaric acid accumulation patterns were similar and closely correlated with the PAL, C4H and POD activities. The patterns of lignin accumulation were closely related to the PAL, POD, and PPO enzymes.

Jasmonate (JA) has a pivotal role in regulating plant developmental programs, including physiological processes and responses to environmental stimuli. In the jasmonate pathway, α-linolenic acid (α-LeA) and hexadecatrienoic acid (HTA) are converted to 12-oxo-phytodienoic acid and dinor OPDA by catalysis with 13-lipoxygenase (13-LOX), alkylene oxide synthase (AOS) and alkylene oxide cyclase (AOC), and then finally converted to JA through an OPR3-independent pathway. JA can bind to Ile catalyzed by JAR1 (JASMONATE RESISTANT 1) to generate JA-Ile (Jasmonoyl-isoleucine), while JA-Ile activates MYC transcription factors by directly binding to JAZ (JASMONATE ZIM DOMAIN 1) and COI1 (COR-insensitive 1), which generates JAZ degradation *via* the 26S proteasome pathway (Wan and Xin, 2022). The results of this study showed that the inoculation with *B. dothidea* upregulated JA pathway-related gene expression (*AcLOX*, *AcAOS AcAOC*, *AcOPR3*, *AcJAR1*, *AcCOI1* and *AcMYC2*) in kiwifruit, and its up-regulation trend was greater in MeJA-treated kiwifruit. Meanwhile, MeJA inhibited JAZ expression, which is a negative gene in the regulation of JA signaling. This further indicated that MeJA effectively enhanced the role of JA pathway in kiwifruit resistance to *B. dothidea*. In studies on sweet cherry (Pan et al., 2022) and peach (Ji et al., 2021), MeJA upregulated the gene expression of *LOX*, *AOS*, *OPR3* and *MYC2* to initiate their defense mechanisms, which was consistent with this study. MYC2 is the master regulator in JA signaling pathway and involved in the regulation of JA-mediated physiological activities in plant (Dombrecht et al., 2007 and Du et al., 2017). It has been reported that MYC2 plays an important role in MeJA-mediated disease resistance in *Arabidopsis thaliana* (Pozo et al., 2008), *Fragaria* × *ananassa* (Valenzuela-Riffo et al., 2020) and *Solanum lycopersicum* (Min et al., 2021). In this work, *AcMYC2* was induced in kiwifruit inoculated with *B. dothidea* and expressed more strongly after MeJA treatment. There was a high positive correlation between *AcMYC2* and total phenolics, lignin as well as phenylpropanoid-related enzyme activities and gene expression in exogenous MeJA-treated kiwifruit. These results indicated that *AcMYC2* may be involved in regulating the MeJA-mediated phenylpropanoid pathway for disease resistance in kiwifruit to against pathogen.

## 5 Conclusions

In summary, MeJA increased the activity of phenylpropanoid pathway-related enzymes (PAL, C4H, 4CL, CAD, POD and PPO) and their gene expression (*AcPAL*, *AcC4H*, *Ac4CL*, and *AcCAD*). Furthermore, MeJA treatment promoted the accumulation of metabolites (total phenolics, flavonoids, chlorogenic acid, caffeic acid and lignin) during the process of kiwifruit resistance to soft rot. Additionally, MeJA regulated the expression of JA pathway-related genes (*AcLOX*, *AcAOS AcAOC*, *AcOPR3*, *AcJAR1*, *AcCOI1*, *AcJAZ* and *AcMYC2*) that resulted in an improved resistance of kiwifruit to the *B. dothidea* pathogen. However, further studies on the mechanism of MeJA-induced kiwifruit against *B. dothidea* in multiomics analyses is needed.

## Data availability statement

The original contributions presented in the study are included in the article/**Supplementary Material**. Further inquiries can be directed to the corresponding author.

## Author contributions

MX and JC conceived the project and designed the experiments. SL, LX, MC, QC, ZL and NK performed the experiments. SL and MJ analyzed the data. SL wrote the manuscript, and MX finalized at last. All authors contributed to the article and approved the submitted version.

## References

[B1] Alim A. Li T. Nisar T. Ren D. Zhai X. Pang Y.. (2019). Antioxidant, antimicrobial, and antiproliferative activity-based comparative study of peel and flesh polyphenols from *Actinidia chinensis* . Food Nutr. Res. 63, 1577. doi: 10.29219/fnr.v63.1577 PMC6495110 31073285

[B2] Carlos C. G. Jean R. Nicolas A. Ludivine D. Sévérine D. Marc F. (2019). Microbial antagonism toward *Botrytis* bunch rot of grapes in multiple field tests using one *Bacillus ginsengihumi* strain and formulated biological control products. Front. Plant Sci. 10. doi: 10.3389/fpls.2019.00105 PMC6378282 30804972

[B3] Chen T. Ji D. C. Zhang Z. Q. Li B. Q. Qin G. Z. Tian S. P. (2021). Advances and strategies for controlling the quality and safety of postharvest fruit. Engineering 7, 1177–1184. doi: 10.1016/j.eng.2020.07.029

[B4] Deng J. J. Yang B. Zhang Z. K. Xie D. F. Ge Y. H. Li W. H.. (2015). Postharvest oxalic acid treatment induces resistance against pink rot by priming in muskmelon (*Cucumis melo* l.) fruit. Postharvest Biol. Technol. 106, 53–61. doi: 10.1016/j.postharvbio.2015.04.005

[B5] Dombrecht B. Xue G. P. Sprague S. J. Kirkegaard J. A. Ross J. J. Reid J. B.. (2007). MYC2 differentially modulates diverse jasmonate-dependent functions in arabidopsis. Plant Cell 19, 2225–2245. doi: 10.1105/tpc.106.048017 17616737 PMC1955694

[B6] Du M. Zhao J. Tzeng D. T. W. Liu Y. Deng L. Yang T.. (2017). MYC2 orchestrates a hierarchical transcriptional cascade that regulates jasmonate-mediated plant immunity in tomato. Plant Cell 29, 1883–1906. doi: 10.1105/tpc.16.00953 28733419 PMC5590496

[B7] Ge Y. H. Tang Q. Li C. Y. Duan B. Li X. Wei M. L.. (2019). Acibenzolar-s-methyl treatment enhances antioxidant ability and phenylpropanoid pathway of blueberries during low temperature storage. LWT-Food Sci. Technol. 110, 48–53. doi: 10.1016/j.lwt.2019.04.069

[B8] Glowacz M. Roets N. Sivakumar. D. (2017). Control of anthracnose disease *via* increased activity of defence related enzymes in ‘Hass’ avocado fruit treated with methyl jasmonate and methyl salicylate. Food Chem. 234, 163–167. doi: 10.1016/j.foodchem.2017.04.063 28551220

[B9] Guo J. Fang W. W. Lu H. P. Zhu R. Y. Lu L. F. Zheng X. D.. (2014). Inhibition of green mold disease in mandarins by preventive applications of methyl jasmonate and antagonistic yeast. Cryptococcus laurentii. Postharvest Biol. Technol. 88, 72–78. doi: 10.1016/j.postharvbio.2013.09.008

[B10] Heleno S. A. Martins A. Queiroz M. J. R. P. Ferreira I. C. F. R. (2015). Bioactivity of phenolic acids: metabolites versus parent compounds: a review. Food Chem. 173, 501–513. doi: 10.1016/j.foodchem.2014.10.057 25466052

[B11] Ji N. N. Wang J. Zuo X. X. Li Y. F. Li M. L. Wang K. T.. (2021). *PpWRKY45* is involved in methyl jasmonate primed disease resistance by enhancing the expression of jasmonate acid biosynthetic and pathogenesis-related genes of peach fruit. Postharvest Biol. Technol. 172, 111390. doi: 10.1016/j.postharvbio.2020.111390

[B12] Li X. Q. Long Y. H. Yin X. H. Wu X. M. Zhao Z. B. Fan R.. (2019c). Mechanism of action of methyl jasmonate against kiwifruit soft rot and its effect on fruit quality. Food Sci. (in Chinese) 40, 239–248. doi: 10.7506/spkx1002-6630-20180625-467

[B13] Li G. L. Meng F. B. Wei X. P. Lin M. (2019a). Postharvest dipping treatment with BABA induced resistance against rot caused by gilbertella persicaria in red pitaya fruit. Scientia Hortic. 257, 108713. doi: 10.1016/j.scienta.2019.108713

[B14] Liu Y. Y. Ge Y. H. Bi Y. Li C. Y. Deng H. W. Hu L. G.. (2014). Effect of postharvest acibenzolar-s-methyl dipping on phenylpropanoid pathway metabolism in muskmelon (*Cucumis melo* l.) fruits. Scientia Hortic. 168, 113–119. doi: 10.1016/j.scienta.2014.01.030

[B15] Livak K. J. Schmittgen T. D. (2001). Analysis of relative gene expression data using real-time quantitative PCR and the 2(-delta delta C(T)) method. Methods 25 (4), 402–408. doi: 10.1006/meth.2001.1262 11846609

[B16] Li S. G. Xu Y. H. Bi Y. Zhang B. Shen S. L. Jiang T. J.. (2019b). Melatonin treatment inhibits gray mold and induces disease resistance in cherry tomato fruit during postharvest. Postharvest Biol. Technol. 157, 110962. doi: 10.1016/j.postharvbio.2019.110962

[B17] Meng X. H. Han J. Wang Q. Tian S. P. (2009). Changes in physiology and quality of peach fruits treated by methyl jasmonate under low temperature stress. Food Chem. 114, 1028–1035. doi: 10.1016/j.foodchem.2008.09.109

[B18] Michailides T. J. Elmer P, A, G. (2000). Botrytis gray mold of kiwifruit caused by *Botrytis cinerea* in the united states and new Zealand. Plant Dis. 84, 208–223. doi: 10.1094/PDIS.2000.84.3.208 30841231

[B19] Min D. D. Li F. J. Zhang X. H. Cui X. X. Shu P. Dong L. L.. (2018). *SlMYC2* involved in MeJA-induced tomato fruit chilling tolerance. J. Agric. Food Chem. 66, 3110–3117. doi: 10.1021/acs.jafc.8b00299 29528226

[B20] Min D. D. Zhou J. X. Li J. Z. Ai W. Li Z. L. Zhang X. H.. (2021). *SlMYC2* targeted regulation of polyamines biosynthesis contributes to methyl jasmonate-induced chilling tolerance in tomato fruit. Postharvest Biol. Technol. 174, 111443. doi: 10.1016/j.postharvbio.2020.111443

[B21] Pan L. Y. Chen X. R. Xu W. Fan S. S. Wan T. Zhang J.. (2022). Methyl jasmonate induces postharvest disease resistance to decay caused by *Alternaria alternata* in sweet cherry fruit. Scientia Hortic. 292, 110624. doi: 10.1016/j.scienta.2021.110624

[B22] Pan L. Y. Zhao X. Y. Chen M. Fu Y. Q. Xiang M. L. Chen J. Y. (2019). Regulation of defense enzymes by methyl jasmonate inducing kiwifruit fruits against soft rot. Plant Prot. (in Chinese) 45, 75–80. doi: 10.16688/j.zwbh.2018172

[B23] Pan L. Y. Zhao X. Y. Chen M. Fu Y. Q. Xiang M. L. Chen J. Y. (2020). Effect of exogenous methyl jasmonate treatment on disease resistance of postharvest kiwifruit. Food Chem. 305, 125483. doi: 10.1016/j.foodchem.2019.125483 31610420

[B24] Pozo M. J. van der Ent S. Van Loon L. C. Pieterse C. M. J. (2008). Transcription factor MYC2 is involved in priming for enhanced defense during rhizobacteria-induced systemic resistance in *Arabidopsis thaliana* . New Phytol. 180, 511–523. doi: 10.1111/j.1469-8137.2008.02578.x 18657213

[B25] Serna-Escolano V. Martínez-Romero D. Giménez M. J. Serrano M. García-Martínez S. Valero D.. (2021). Enhancing antioxidant systems by preharvest treatments with methyl jasmonate and salicylic acid leads to maintain lemon quality during cold storage. Food Chem. 338, 128044. doi: 10.1016/j.foodchem.2020.128044 32932092

[B26] Takshak S. Agrawal S. B. (2014). Secondary metabolites and phenylpropanoid pathway enzymes as influenced under supplemental ultraviolet-b radiation in *Withania somnifera* dunal, an indigenous medicinal plant. J. Photochem. Photobiol. B: Biol. 140, 332–343. doi: 10.1016/j.jphotobiol.2014.08.011 25226342

[B27] Tzortzakis N. Chrysargyris A. Sivakumar D. Loulakakis K. (2016). Vapour or dipping applications of methyl jasmonate, vinegar and sage oil for pepper fruit sanitation towards grey mould. Postharvest Biol. Technol. 118, 120–127. doi: 10.1016/j.postharvbio.2016.04.004

[B28] Valenzuela-Riffo F. Zúñiga P. E. Morales-Quintana L. Lolas M. Cáceres M. Figueroa C. R. (2020). Priming of defense systems and upregulation of *MYC2* and *JAZ1* genes after *Botrytis cinerea* inoculation in methyl jasmonate-treated strawberry fruits. Plants 9, 447. doi: 10.3390/plants9040447 32252456 PMC7238239

[B29] Wang C. W. Ai J. Lv H. Y. Qin H. Y. Yang Y. M. Liu Y. X.. (2015a). First report of penicillium expansum causing postharvest decay on stored kiwifruit (*Actinidia arguta*) in China. Plant Dis. 99, 1037. doi: 10.1094/PDIS-12-14-1274-PDN

[B30] Wang K. T. Jin P. Cao S. F. Shang H. T. Yang Z. F. Zheng Y. H. (2009). Methyl jasmonate reduces decay and enhances antioxidant capacity in chinese bayberries. J. Agric. Food Chem. 57, 5809–5815. doi: 10.1021/jf900914a 19522511

[B31] Wang L. Jin P. Wang J. Jiang L. L. Shan T. M. Zheng Y. H. (2015b). Methyl jasmonate primed defense responses against penicillium expansum in sweet cherry fruit. Plant Mol. Biol. Rep. 33, 1464–1471. doi: 10.1007/s11105-014-0844-8

[B32] Wang H. B. Kou X. H. Wu C. E. Fan G. J. Li T. T. (2020a). Methyl jasmonate induces the resistance of postharvest blueberry to gray mold caused by *Botrytis cinerea* . J. Sci. Food Agric. 100, 4272–4281. doi: 10.1002/jsfa.10469 32378217

[B33] Wang H. B. Wu Y. Yu R. P. Wu C. E. Fan G. J. Li T. T. (2019). Effects of postharvest application of methyl jasmonate on physicochemical characteristics and antioxidant system of the blueberry fruit. Scientia Hortic. 258, 108785. doi: 10.1016/j.scienta.2019.108785

[B34] Wang Y. Xiong G. He Z. Yan M. Zou M. Jiang J. (2020b). Transcriptome analysis of *Actinidia chinensis* in response to *Botryosphaeria dothidea* infection. PloS One 15, e0227303. doi: 10.1371/journal.pone.0227303 31914162 PMC6948751

[B35] Wang C. Zhang J. Xie J. M. Yu J. H. Li J. Lv J.. (2022). Effects of preharvest methyl jasmonate and salicylic acid treatments on growth, quality, volatile components, and antioxidant systems of Chinese chives. Front. Plant Sci. 12. doi: 10.3389/fpls.2021.767335 PMC8777190 35069623

[B36] Wan S. W. Xin X. F. (2022). Regulation and integration of plant jasmonate signaling: a comparative view of monocot and dicot. J. Genet. Genomics, 49, 704–714. doi: 10.1016/j.jgg.2022.04.002 35452856

[B37] Wei X. B. Guan W. L. Yang Y. J. Shao Y. L. Mao L. C. (2021). Methyl jasmonate promotes wound healing by activation of phenylpropanoid metabolism in harvested kiwifruit. Postharvest Biol. Technol. 175, 111472. doi: 10.1016/j.postharvbio.2021.111472

[B38] Wei Y. Y. Zhou D. D. Peng J. Pan L. Q. Tu K. (2017). Hot air treatment induces disease resistance through activating the phenylpropanoid metabolism in cherry tomato fruit. J. Agric. Food Chem. 65, 8003–8010. doi: 10.1021/acs.jafc.7b02599 28813608

[B39] Xu J. Zhang Z. Li X. P. Wei J. Wu B. (2019). Effect of nitrous oxide against *Botrytis cinerea* and phenylpropanoid pathway metabolism in table grapes. Scientia Hortic. 254, 99–105. doi: 10.1016/j.scienta.2019.04.061

[B40] Yang Y. Yang X. H. Guo X. Hu X. X. Dong D. F. Li G. C.. (2022). Exogenously applied methyl jasmonate induces early defense related genes in response to *Phytophthora infestans* infection in potato plants. Hortic. Plant J. 8, 511–526. doi: 10.1016/j.hpj.2022.04.003

[B41] Yao H. J. Tian S. P. (2005). Effects of pre- and post-harvest application of salicylic acid or methyl jasmonate on inducing disease resistance of sweet cherry fruit in storage. Postharvest Biol. Technol. 35, 253–262. doi: 10.1016/j.postharvbio.2004.09.001

[B42] Yue J. Liu J. Tang W. Wu Y. Q. Tang X. Li W.. (2020). Kiwifruit genome database (KGD): a comprehensive resource for kiwifruit genomics. Horticulture Res. 7, 117. doi: 10.1038/s41438-020-0338-9 PMC7395147 32821400

[B43] Yu X. X. Zhang W. J. Zhang Y. Zhang. X. J. Lang D. Y. Zhang X. H. (2019). The roles of methyl jasmonate to stress in plants. Funct. Plant Biol. 46, 197–212. doi: 10.1071/FP18106 32172764

[B44] Zhang Y. Z. Li P. M. Cheng L. L. (2010). Developmental changes of carbohydrates, organic acids, amino acids, and phenolic compounds in ‘Honeycrisp’ apple flesh. Food Chem. 123, 1013–1018. doi: 10.1016/j.foodchem.2010.05.053

[B45] Zhang M. Y. Wang D. J. Gao X. X. Yue Z. Y. Zhou H. L. (2020). Exogenous caffeic acid and epicatechin enhance resistance against *Botrytis cinerea* through activation of the phenylpropanoid pathway in apples. Scientia Hortic. 268, 109348. doi: 10.1016/j.scienta.2020.109348

[B46] Zhou Y. Gong G. S. Cui Y. L. Zhang D. X. Chang X. L. Hu R. P.. (2015). Identification of *Botryosphaeriaceae* species causing kiwifruit rot in sichuan province, China. Plant Dis. 99, 699–708. doi: 10.1094/PDIS-07-14-0727-RE 30699681

[B47] Zhou F. H. Jiang A. L. Feng K. Gu S. T. Xu D. Y. Hu W. Z. (2019). Effect of methyl jasmonate on wound healing and resistance in fresh-cut potato cubes. Postharvest Biol. Technol. 157, 110958. doi: 10.1016/j.postharvbio.2019.110958

[B48] Zhuo R. L. Li B. Q. Tian S. P. (2022). Alginate oligosaccharide improves resistance to postharvest decay and quality in kiwifruit (*Actinidia deliciosa* cv. Bruno). Hortic. Plant J. 8, 44–52. doi: 10.1016/j.hpj.2021.07.003

