# Supplementary material for: The involvement of the phenylpropanoid and jasmonate pathways in methyl jasmonate-induced soft rot resistance in kiwifruit (Actinidia chinensis)
Source: Front Plant Sci. 2022 Dec 16;13:1097733. doi: 10.3389/fpls.2022.1097733 (PMC9800925; doi:10.3389/fpls.2022.1097733)
Supplement: Supplementary file 1 [file Table_1.docx]

Supplementary Material

The involvement of the phenylpropanoid and jasmonate pathways in methyl jasmonate-induced soft rot resistance in kiwifruit (*Actinidia chinensis*)

Shucheng Li, Liuhua Xiao, Ming Chen, Qing Cao, Zhenyu Luo, Naihui Kang, Mingshu Jia, Jinyin Chen, Miaolian Xiang*

*****Corresponding Author: mlxiang2010@126.com

**Table S1** Sequences of primers used qRT-PCR analysis.

| **Gene** | **Accession number** | **Primer sequence 5’→3’** |
| --- | --- | --- |
| *AcActin* (actin) | Ach05g107181 | F: GCTTACAGAGGCACCACTCAACC  R: CCGGAATCCAGCACAATACCAG |
| *AcPAL* (phenylalanine ammonialyase) | Actinidia13078 | F: TCTCCTCCAGGGTTACTCCG  R: GCATTTAGGGTTTCCCCGGT |
| *AcC4H* (cinnamate 4-hydroxylase) | Ach01g022871 | F: CGGTTCTTGGAGGAGGATGG  R: CAATCTTCTCCGGCCCTTGT |
| *Ac4CL* (4-coumarate--CoA ligase) | Actinidia18707 | F: AATCACCCCTACCCCTCCTC  R: TTCTGTCGGGGAATTGGGTG |
| *AcCAD* (cinnamyl alcohol dehydrogenase) | Actinidia28817 | F: CCGACCTCCACCAGATCAAG  R: CACCCAACCCCAACTCTGTC |
| *AcAOS* (allene oxide synthase) | Actinidia29849 | F: TCTCCTATCTCGACCCGTCC  R: ACCGGCGTTTCCATTTTTGG |
| *AcAOC* (allene oxide cyclase) | Ach27g170451.2 | F: TATGCGTTCTCATCCAGCAC  R: TCTGCTGCAGTTTCACCTCG |
| *AcOPR3* (12-oxophytodienoate reductase 3) | Ach10g085821.2 | F: GACGAGAAGGTGATCCGACC  R: AATCCTCCAACAGCTCCGTG |
| *AcJAR1* (jasmonate resistant 1) | Actinidia10109 | F: CTTGAGCGAAGCACACCATC  R: GCAAGTTCTACTCGCGCAAC |
| *AcCOI1* (coronatine insensitive 1) | Actinidia13918 | F: GGTCCTGGAATCGGACTTGG  R: CCGCGTTGCAATACCTGAAG |
| *AcJAZ* (jasmonate ZIM domain) | Actinidia30242 | F: CGCGGTTTGGAAGGAAATGG  R: GGGTCGGAATGACCGAAAGT |
| *AcMYC2* (transcription factor MYC2) | Actinidia07315 | F: TTGTGGGGACGTTAGGGATG  R: CGAGGAGGAGGGATGAGAGA |
| *AcLOX* (lipoxygenase) | Ach23g115201 | F: TCCAAGACGAGCTCCTGAAG  R: CTGGTGGGAACACTGTGAGC |

Note: Gene accession numbers were identified from the Cornell University kiwifruit genome database (http://kiwifruitgenome.org/).
